# Effect of methylphenidate on oculomotor function in individuals with multiple sclerosis: a pilot randomized placebo-controlled trial

**DOI:** 10.3389/fneur.2024.1393877

**Published:** 2024-05-23

**Authors:** Timothy J. Rich, Aubree Alexander, Ekaterina Dobryakova, Nancy D. Chiaravalloti, John DeLuca, Silvana L Costa

**Affiliations:** ^1^Center for Stroke Rehabilitation Research, Kessler Foundation, West Orange, NJ, United States; ^2^Department of Physical Medicine Rehabilitation, Rutgers New Jersey Medical School, Newark, NJ, United States; ^3^Center for Neuropsychology and Neuroscience Research, Kessler Foundation, East Hanover, NJ, United States; ^4^Center for Traumatic Brain Injury Research, Kessler Foundation, East Hanover, NJ, United States; ^5^Center for Multiple Sclerosis Research, Kessler Foundation, West Orange, NJ, United States

**Keywords:** multiple sclerosis, processing speed, methylphenidate, oculomotor muscles, eye movements

## Abstract

**Introduction:**

Individuals with multiple sclerosis (MS) frequently experience visual and oculomotor symptoms that may impact and confound neuropsychological assessments of information processing speed (IPS). In this study, we examined the effect of the psychostimulant methylphenidate on oculomotor function and the association between change in oculomotor speed and change in information processing speed.

**Methods:**

We used a repeated measures crossover design in which a sample of 11 participants with MS were randomly assigned to one of two treatment arms: one that received methylphenidate for 4 weeks and another that received a placebo for 4 weeks. After a 7-day washout period, the treatments were crossed over. The King Devick test, the Symbol Digit Modalities Test, and the Paced Auditory Serial Addition Test were administered at baseline and after each of the two study arms.

**Results:**

We found a significant improvement in oculomotor speed in the methylphenidate condition as compared to placebo. This improvement was significantly correlated with improvement on a visuomotor assessment of IPS (Symbol Digit Modalities Test), but no such association was found for an auditory-verbal assessment of IPS (Paced Auditory Serial Addition Test).

**Discussion:**

These findings suggest that individuals with MS experience improved oculomotor speed while taking methylphenidate, which may, in turn, improve performance on assessments of IPS with visuomotor demands.

## 1 Introduction

Multiple sclerosis (MS) is a demyelinating neurologic disease characterized by a range of cognitive and sensorimotor symptoms, including reduced information processing speed (IPS) (1), fatigue, and visual and oculomotor dysfunction (2). Visual symptoms include blurry vision, poor acuity, and accommodative dysfunction (3). Oculomotor symptoms include strabismus, internuclear ophthalmoplegia, nystagmus, saccadic hypermetria, saccadic oscillations, inhibition of the vestibulo-ocular reflex, and impaired vergence, stereopsis, and smooth pursuit (4). Furthermore, individuals with MS frequently report symptoms of visual discomfort and oculomotor fatigue (5).

Typically, individuals with MS experience intermittent attacks of acute neuroinflammation followed by full or partial recovery. Over time, many with MS experience a progressive neurodegenerative process secondary to the cumulative effects of these acute episodes (3). Visual and oculomotor symptoms are no exception: long-term deficits are common even when clinical recovery from an acute episode is achieved (2).

The King-Devick (K-D) test (6) is used for detecting oculomotor inefficiencies associated with sports-related concussion with high sensitivity and specificity via a rapid automatized naming task (7). The K-D has been used extensively in both clinical care and research for concussion and has recently been used in other neurological populations, including MS. Individuals with MS present with slower K-D performance than healthy controls, which is associated with visual dysfunction, neurologic disability, and reduced vision-specific quality of life (8–10).

Methylphenidate is a psychostimulant medication shown to be effective in reducing impulsivity, inattentiveness, and hyperactivity in individuals with attention deficit hyperactivity disorder (ADHD) (11). In addition to its general effects on arousal and attention, methylphenidate has been shown to improve visual and/or oculomotor function in those with ADHD as well as healthy participants (11). Methylphenidate has also been used to treat symptoms of multiple neurologic conditions including fatigue in MS (12).

The current study is a secondary analysis of data from a pilot randomized clinical trial that examined the efficacy of methylphenidate on self-reported fatigue in MS (12). The objectives of the current analyses were to (a) examine the specific impact of methylphenidate on oculomotor function via performance on the K-D and (b) determine if improvements on the K-D are associated with improvements in IPS via the Symbol Digit Modalities Test (SDMT) (13) and the Paced Auditory Serial Addition Test (PASAT) (14).

## 2 Materials and methods

### 2.1 Participants

Twelve English-speaking participants with clinically definite MS were recruited from the Kessler Foundation MS research participant database, local support groups for individuals with MS, and referrals from physicians from the MS clinic at University Hospital in Newark, New Jersey. Participants were excluded if they (a) had a history of alcohol or drug abuse; (b) were taking corticosteroids or psychostimulants at the time of enrollment; (c) consumed more than 300 milligrams of caffeine per day; (d) had a history of neurological disorder other than MS; or (e) had a diagnosis of ADHD, depression, thyroid disease, anemia, or low vitamin D. This study conforms with the Declaration of Helsinki and was approved by the Kessler Foundation Institutional Review Board. All participants completed the informed consent process prior to enrollment.

Of the 12 participants recruited for this study, one dropped out of the study during the washout period, following completion of study arm I (placebo) but prior to beginning study arm II (methylphenidate), leaving 11 participants (median age: 60 [IQR: 52–62]; 81.8% female; median years education: 16 [IQR: 15–16]; *N* = 7 with relapsing–remitting type MS, *N* = 4 with progressive type). Participants were provided access to a telephone hotline, available 24 h per day, to ask questions related to the study or to report any perceived side effects of medication.

### 2.2 Procedure

After enrollment, participants were randomly assigned to one of two treatment arms. In one arm, participants were administered methylphenidate (20 mg/day in the morning, immediate release) for 4 weeks, and in the other participants were administered a placebo (20 mg/day in the morning) for 4 weeks. This dosage was determined based on prior literature (15–18). After a 7-day washout period, the treatments were crossed over so that those who took methylphenidate were administered the placebo and vice versa. Participants completed assessments of physical, cognitive, and psychosocial function at 3 timepoints: baseline, and after each of the two study arms. Assessment sessions were approximately 2 h in duration and were scheduled in the morning to minimize effects of fatigue progression over the course of the day. All investigators were blinded to which treatment arm had been completed prior to testing.

### 2.3 Data analysis

IBM SPSS Statistics v26 was used. A one-way repeated measures ANOVA compared means between the three time points and *post hoc* pairwise comparisons were conducted using the Bonferroni correction. To determine the relationship between changes on the measures from baseline to the methylphenidate condition, Pearson correlations were performed. Alpha was set to 0.05 for all analyses.

### 2.4 Outcome measures

Participants completed the K-D, the SDMT, and the PASAT as part of the larger neuropsychological battery conducted at 3 study timepoints described above. The K-D is a measure of oculomotor speed. In the K-D, participants are instructed to read aloud numbers presented on an 8.5″ × 11″ card as quickly as possible. The test consists of three stimulus cards with identical set sizes (5 numbers on 8 rows) but progressively increasing visual complexity, and, thus, difficulty. Numbers on the first card are spread out and connected by horizontal lines to guide eye movements. Numbers on the second card are spread out in a similar manner to the first card but are not connected by lines. Numbers on the third card are more condensed, with less vertical spacing between rows. Errors and time to read each card are recorded. The final K-D score is derived from the sum of the time to complete each of the three test cards (8).

The SDMT is considered to be the gold standard screening tool for detection of cognitive dysfunction in MS (19). The SDMT is a paper-and-pencil task in which participants pair a series of geometric symbols with numbers by following a reference key located at the top of the page. The oral version was administered to eliminate the influence of upper extremity motor limitations on test performance. Participants are given 90 s to say the corresponding number for each symbol provided. The final SDMT score is the number of correct responses within the 90 s timeframe.

The PASAT is a serial addition task used frequently in MS to measure auditory IPS, working memory, and attention (1). On each trial, a series of single digit numbers is read aloud to the participant at fixed intervals. The participant’s task is to verbally respond with the sum of the two most recently read numbers prior to the presentation of the next digit. The version used in this study consists of 2 trials with different speed of stimulus presentation: 2 and 3 s. We used the total number of correct responses on the 3-s trial as the final PASAT score. Of note, one participant in our sample was missing PASAT data for all three timepoints so was excluded from analyses using this variable. Another participant was missing data for the placebo condition only, which was imputed via the last observation carried forward method.

## 3 Results

**Figure 1:**
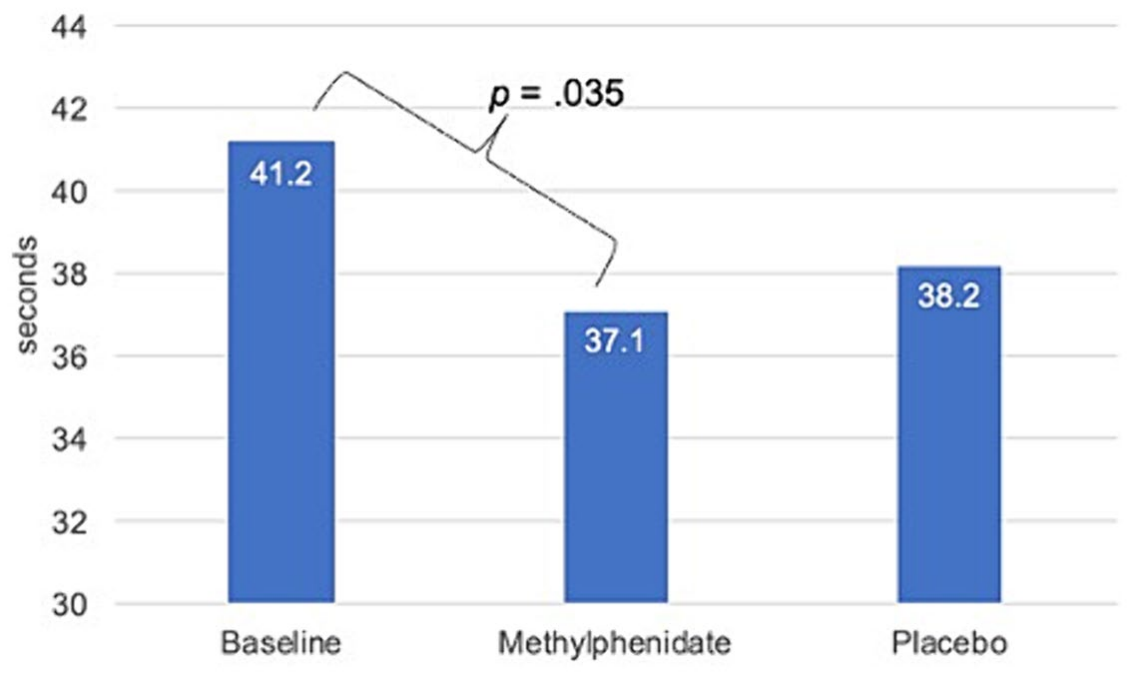
Mean time on King-Devick test at baseline, after methylphenidate, and after placebo.

The mean time to complete the K-D differed significantly between conditions [*F*(2, 20) = 4.67, *p* = 0.022] with a large effect size (η^2^ = 0.32). The pairwise comparison showed a significant reduction in mean K-D time in the methylphenidate condition as compared to the baseline condition (37.1 [SE = 3.6] vs. 41.2 [SE = 4.1] seconds, respectively, *p* = 0.035, Cohen’s *d* = 0.3; see Figure 1). No significant reduction was found for mean time to complete the K-D in the placebo condition (38.2 [SE = 3.2] seconds) as compared to baseline (*p* = 0.250) and no significant difference was found in the number of errors between the baseline and methylphenidate conditions (*p* = 0.625).

We did not find a significant difference in SDMT scores [*F*(2,20) = 0.94, *p* = 0.407]. However, a series of Pearson correlations showed a strong association between change on the K-D and change on the SDMT (*r^2^* = −0.80, *p* = 0.003). We found significant differences for the PASAT [*F*(2,18) = 5.77, *p* = 0.012] with a *post hoc* pairwise comparison using the Bonferroni correction showing a marginally significant improvement in PASAT scores in the methylphenidate condition as compared to the baseline condition (43.7 ± 4.0 vs. 38.7 ± 4.9, respectively, *p* = 0.053). No significant differences were found for the PASAT in the placebo condition (41.2 ± 4.4) as compared to baseline (*p* = 0.245). No association was found between change on the K-D and change on the PASAT (*r^2^* = 0.14, *p* = 0.695). One participant reported visual symptoms but continued in the study after medical consultation determined the symptoms were unrelated to the study.

## 4 Discussion

In this pilot randomized controlled trial, we used a crossover design to examine the effects of methylphenidate on performance on the K-D in 11 participants with clinically definite MS. We found that participants had significantly improved K-D scores while taking methylphenidate with no significant change in K-D scores in the placebo condition. We found that change on the K-D strongly correlated with change on the SDMT, which assesses visuomotor IPS, but did not correlate with change on the PASAT, which assesses auditory IPS and working memory. These results are consistent with past research that found that the K-D explained 31% of the variance in the SDMT (20). In addition, our finding that performance on the K-D improved while taking methylphenidate is consistent with prior studies that have demonstrated improved visual processing speed and oculomotor function in both children with ADHD and healthy adults when taking methylphenidate (11).

Deficits of MS, such as cognitive dysfunction and reduced IPS, are traditionally captured by neuropsychological measures specific to these domains. However, it remains unclear if performance on these measures is predicted or confounded by deficits in other cognitive domains (3, 20, 21). The interdependence of cognitive deficits in various domains and their impact on one another is not well understood.

According to the tri-factor model of IPS in MS (1), the construct of IPS is reliant on cognitive speed, sensorial speed (e.g., visual processing speed), and motor speed (e.g., oculomotor function). The SDMT involves all three factors of the tri-factor model. Thus, it is sensitive to detect change in MS but is insufficient to determine the granular factors that underlie performance and performance deficits (1, 22). Prior research from our group found an association between MS-related oculomotor dysfunction and poorer performance on the SDMT, which suggests that the oculomotor demands of the task may influence the cognitive demands of the task. Our finding that improvements on the K-D between the baseline and experimental conditions correlated with improvements on the SDMT is consistent with this and other prior studies that explored visual and oculomotor correlates of SDMT performance (3, 20, 21).

In contrast to the SDMT, improvements on the K-D did not correlate with change on the PASAT, which, in addition to working memory and attention, emphasizes auditory rather than visual IPS (23). Although all of these domains are affected by MS, deficits in IPS have been shown to underlie poor performance on the PASAT (24). In the context of the tri-factor model of IPS in MS, our findings suggest that methylphenidate improved oculomotor speed rather than cognitive or sensorial speed in our sample.

There are important limitations to this study that should be considered. First, this was secondary analysis of a pilot randomized clinical trial, so the sample was small. Thus, more robust analyses of mediation and moderation were not possible. Second, we relied on the final score of the K-D to frame our analyses. Although the K-D is considered to be a measure of oculomotor function, many disparate neurocognitive functions other than oculomotility could be involved. Therefore, future studies examining the impact of impaired visual or oculomotor function to the overall functional picture of individuals with multiple sclerosis should include oculography and/or detailed ophthalmometric examination.

In conclusion, participants with MS showed improved oculomotor function while taking methylphenidate as compared to placebo. In addition, this improvement was associated with improved IPS as measured by the SDMT, which has visuomotor demands; but was not associated with improved IPS as measured by the PASAT, which has auditory-verbal demands. Thus, methylphenidate may be beneficial for individuals with MS who experience oculomotor deficits and may indirectly promote improved processing speed for visuomotor tasks.

## Data availability statement

The raw data supporting the conclusions of this article will be made available by the authors, without undue reservation.

## Ethics statement

The studies involving humans were approved by Kessler Foundation Institutional Review Board. The studies were conducted in accordance with the local legislation and institutional requirements. The participants provided their written informed consent to participate in this study.

## Author contributions

TR: Writing – review & editing, Writing – original draft, Formal analysis, Conceptualization. AA: Writing – review & editing, Writing – original draft. ED: Writing – review & editing, Investigation, Data curation. NC: Writing – review & editing, Supervision. JD: Writing – review & editing, Funding acquisition. SLC: Writing – review & editing, Writing – original draft, Supervision, Investigation, Formal analysis, Conceptualization.
